# Functional diversity dynamics of waterbird communities driven by water levels at Shengjin Lake, a small river‐connected shallow lake in the middle and lower Yangtze River floodplain

**DOI:** 10.1002/ece3.70222

**Published:** 2024-10-07

**Authors:** Yongzhi Wang, Xianglin Ji, Lizhi Zhou

**Affiliations:** ^1^ School of Resources and Environmental Engineering Anhui University Hefei China; ^2^ Anhui Province Key Laboratory of Wetland Ecosystem Protection and Restoration (Anhui University) Hefei China; ^3^ Anhui Shengjin Lake Wetland Ecology National Long‐Term Scientific Research Base Dongzhi China

**Keywords:** functional diversity, habitat filtering, hydrological dynamics, Shengjin Lake, Waterbird community

## Abstract

Gate‐controlled activities in lakes can directly or indirectly influence the assembly of waterbird communities. Shengjin Lake, a Ramsar site, is a typical river‐connected and gate‐controlled shallow lake in the lower and middle Yangtze River floodplain in China, comprising three sub‐lakes (upper, middle, and lower) based on topographical features. We surveyed wintering waterbirds at Shengjin Lake from October 2022 to March 2023. We divided the winter water level period into nine phases based on the characteristics of water level changes. By measuring functional diversity, we aimed to provide insights into the differences in waterbird communities among the three sub‐lakes under different water level conditions. Multiple linear regression was used to analyze the relationship between habitat factors and functional diversity. We further explored the relationship between specific functional traits and habitat factors through a combination of the R‐mode linked to the Q‐mode and the trait‐environment correlation matrix (fourth‐corner analyses) to explain the mechanism underlying waterbird community assembly. When the water level fluctuated in the range of 10.43–10.74 m (Huanghai elevation), the three sub‐lakes had significant habitat differences and high habitat heterogeneity, increasing functional richness and functional dispersion of the upper and lower lakes, both of which significantly differed from those of the middle lake. Habitat heterogeneity and mudflat habitats have positive effects on functional diversity. The difference in functional diversity was primarily determined based on the foraging traits and strata of waterbirds. Habitat filtering of particular traits is a major driving force underlying the assembly of waterbird communities. Overall, we suggest that the minimum water level in the wintering period at Shengjin Lake should be regulated between 10.43 and 10.74 m. These findings provide reasonable suggestions for water level regulation and a theoretical basis for conserving waterbird diversity at Shengjin Lake.

## Graphical abstract

The study is based on the perspective of functional diversity to investigate the impact of water level changes on waterbird community diversity. Changes in habitat heterogeneity are the main cause of functional diversity differences. Habitat filtering is identified as the primary driver shaping the waterbird community assembly.

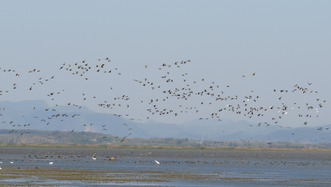

## 1 INTRODUCTION

Understanding the ecological processes involved in biodiversity and community organization is essential for community ecology research (Gotzenberger et al., 2012). The diversity of waterbird communities reflects wetland ecosystem structure, function, and ecological processes and is indicative of wetland ecological conditions (Bonthoux & Balent, 2015). Waterbirds have diverse biological characteristics and engage in complex resource utilization behaviors, making them useful indicator species in wetland ecosystems (Moreno‐Opo et al., 2011). Community diversity of waterbirds is influenced by various factors, such as food resources (Bai et al., 2021), habitat size and structure (Zhou et al., 2020), water level changes (Krajewski et al., 2023), and human disturbances (Rosin et al., 2012). Among those factors, water level changes are particularly important because habitat conditions for waterbirds are largely determined by changes in water levels (Larson et al., 2020). Therefore, studying the effects of water level conditions on waterbird community diversity through habitat mediation will help develop effective strategies for conserving waterbirds.

The middle and lower Yangtze River floodplain has the densest lake distribution in China. Based on hydrological conditions, the lakes are divided into natural river‐connected and gate‐controlled river‐connected lakes. Under the influence of natural hydrological rhythms, seasonal water level fluctuations lead to high heterogeneity in lake habitats, providing suitable habitats for waterbirds and attracting millions of wintering waterbirds (Wang et al., 2017). Anthropogenic regulation of water levels through gates is commonly observed in floodplain lakes to ensure safety for human settlements and livelihoods. The hydrological rhythms of lakes during winter are gate‐controlled, resulting in substantial differences in habitats between different lake regions. When water levels are high, lake habitats tend to homogenize, and when water levels decline, habitat heterogeneity increases as different separate habitats are formed (Li, Yang, et al., 2019).

Habitat filtering allows waterbirds with similar adaptive functional traits to congregate in specific habitats and form specific community structures (Lebrija‐Trejos et al., 2010). The assembly of such communities is also affected by changes in hydrographic rhythms, which directly or indirectly affect the abundance and availability of food resources for waterbirds. Different hydrological conditions in different phases and regions attract different waterbirds, forming diverse communities (Bai et al., 2021). Thus, changes in water levels are a major factor affecting wintering waterbird communities, with different hydrological conditions leading to heterogeneous habitats (Wei & Zhou, 2023). Rational water level regulation might help maintain waterbird community diversity (Ozgencil et al., 2020; Zhang, Zhou, et al., 2021).

Community diversity objectively indicates community species composition and relative abundance (Zellweger et al., 2017). Ecological community research tends to focus on changes in taxonomic diversity (Hovick et al., 2015), not the ecosystem function of species (Tscharntke et al., 2012). An increasing number of ecologists have suggested considering and quantifying other aspects of biodiversity is vital to understanding the processes of community assembly and maintenance. With the accumulation of avian functional trait data, research on community assembly and maintenance processes from the perspective of functional diversity has been increasing (Li, Zhang, et al., 2019). Functional diversity reflects the diversity and distribution of functional traits, effectively explains ecosystem functions, processes, and community composition, and is a measure of the diversity of species characteristics in a community (Orlandi Laureto et al., 2015). Therefore, on the basis of functional diversity, researchers can more comprehensively analyze community assembly and dynamics and reveal the effects of ecological processes, such as habitat filtering and similarity constraints, on waterbird communities (Daniel et al., 2019).

Shengjin Lake is a typical gate‐controlled shallow river‐connected lake in the middle and lower Yangtze River floodplain. This lake is a wintering and migratory stopover site for waterbirds on the East Asian–Australasian Flyway (Fan et al., 2020), and significant changes in water level occur during the wintering period. The lake comprises three sub‐lakes (upper lake, UL; middle lake, ML; and lower lake, LL) based on their topographic features and water flow direction (Li, Yang, et al., 2019). Because the bed of the ML is lower than that of the UL and LL, the decrease in water levels during the wintering period leads to differences in habitat characteristics, with the UL and LL gradually exposing large areas of mudflats and meadows, and the ML habitat remaining submerged.

In this study, we aimed to quantify the habitat differences, including functional diversity differences, among the three sub‐lakes at different water levels and to determine the factors leading to these differences. Overall, we proposed three hypotheses: (1) Habitat heterogeneity in the three sub‐lakes at different water level phase results in differences in the functional diversity of waterbird communities; (2) habitat filtering on the functional traits of specific waterbirds results in differences in functional diversity; and (3) at the lowest water level phase, the functional diversity of waterbird communities in the three sub‐lakes is the highest, and the differences are the greatest.

## 2 MATERIALS AND METHODS

### 2.1 Study area

**FIGURE 1:**
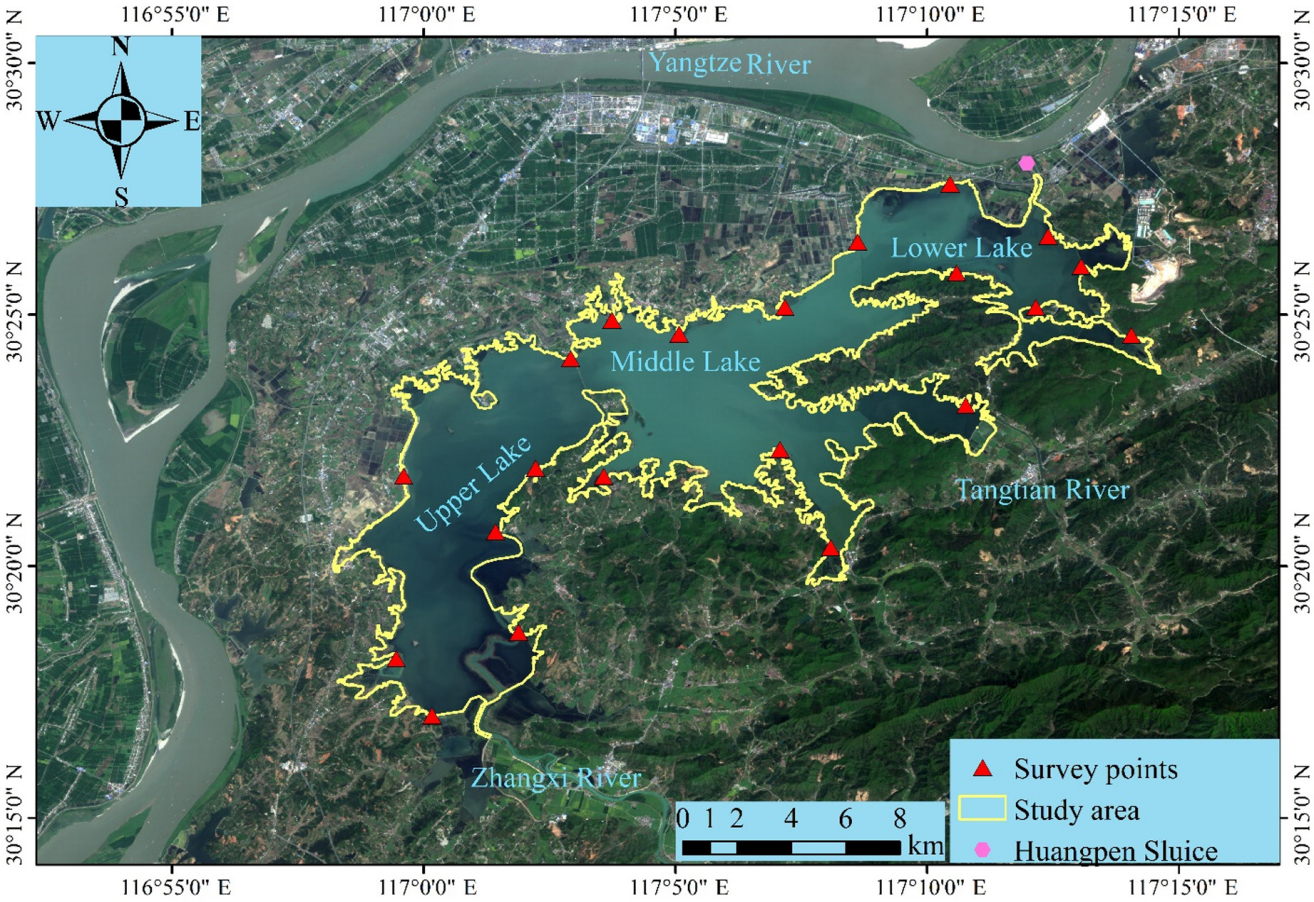
Study area and sample sites at Shengjin Lake, China.

Shengjin Lake (116°55′–117°15′ E, 30°15′–30°30′ N) is located on the right bank of the middle and lower Yangtze River in China (Figure 1), with a subtropical monsoon climate and four distinct seasons. Annually, the water level of the lake decreases in the dry season in October every year and then gradually increases in February of the following year.

The terrain of Shengjin Lake is high in the west and low in the east. The UL runs from Zhangxi Town in the north to the Xiaoluzui Bridge. The ML spans from the east of the bridge to the southwestern part of Babaizhang. The northern end of Babaizhang to Gangyao is the LL. The UL receives inflow from several rivers, including the Zhangxi River, and the Tangtian River flows into the ML. The lake water flows into the Yangtze River from the Huangpen River through the LL, and the water level at the Huangpen Sluice is used to represent that of Shengjin Lake. In autumn and winter, the Huangpen Sluice is opened, leading to a decrease in the water level. When the water level starts to decrease, parts of Shengjin Lake are connected to form a deep‐water habitat. As the water levels decrease during the dry season, the three sub‐lakes are relatively separate and show different habitat characteristics. The habitats of the UL and LL mainly consist of meadows and mudflats, whereas the ML is dominated by water habitats throughout the wintering period. In 2015, Shengjin Lake was listed as a Ramsar site because it provides a habitat for a large number of migratory waterbirds every year. During our surveys, more than 50 species of waterbirds wintered there, among which were species listed on the IUCN Red List, such as the Hooded Crane (*Grus monacha*), Siberian Crane (*Leucogeranus leucogeranus*), Oriental Stork (*Ciconia boyciana*), and Lesser White‐fronted Goose (*Anser erythropus*).

### 2.2 Data collection

#### 2.2.1 Water level

**FIGURE 2:**
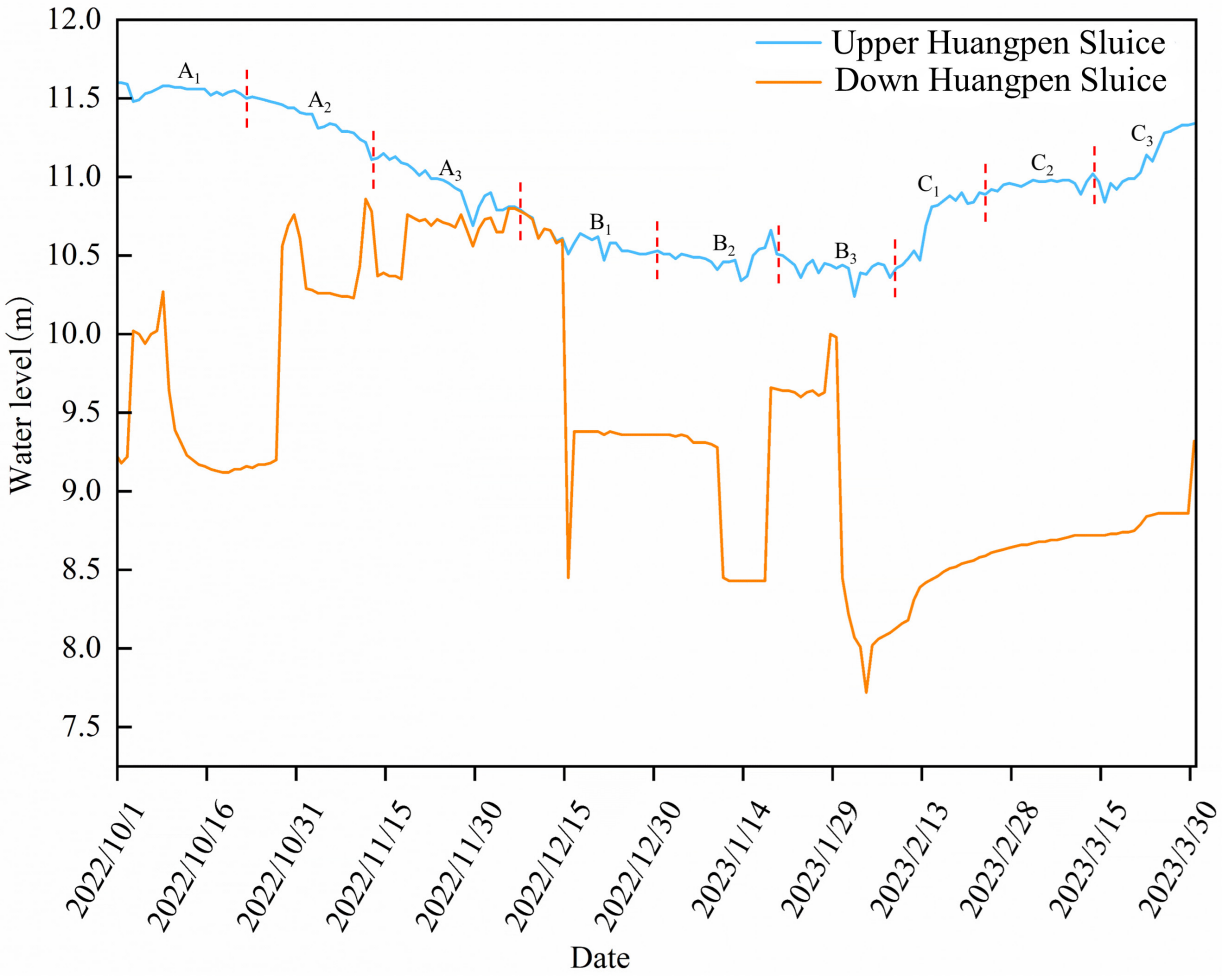
Changes in water level at Huangpen Sluice during the 2022–2023 wintering period (A_1_: Early phase of decline, A_2_: Middle phase of decline, A_3_: Late phase of decline, B_1_: Early phase of stability, B_2_: Middle phase of stability, B_3_: Late phase of stability, C_1_: Early phase of rebound, C_2_: Middle phase of rebound, C_3_: Late phase of rebound).

The Huangpen Sluice controls the connectivity between Shengjin Lake and the Yangtze River. The level at the lower part of the Huangpeng Sluice represents the water level of the Yangtze River, which is determined by the normal hydrological rhythm. The level at the upper part of the Huangpeng Sluice represents the water level of Shengjin Lake, which is determined by a controlled hydrological rhythm. Therefore, water level data (Huanghai elevation) from the upper part of the Huangpen gate were selected to represent the water level of Shengjin Lake. Water level data were obtained from the hydrological information network of Anhui Province (http://yc.wswj.net/ahsxx/lol/public/public.html). Daily water level data were collected from October 2022 to March 2023 at 08:00. The water level of Shengjin Lake showed a decline–stable–rebound trend during the dry season (Figure 2). Based on water level changes, we divided the entire wintering period of waterbirds into three phases: (A) a declining water level from early October to mid‐December, (B) a relatively stable water level from mid‐December to early February, and (C) the rebound of the water level from early February to late March. The phases of water level changes were divided into nine sub‐phases: (A_1_) early, (A_2_) middle, and (A_3_) late water level decline; (B_1_) early, (B_2_) middle, and (B_3_) late water level stability; and (C_1_) early, (C_2_) middle, and (C_3_) late water level rebound.

#### 2.2.2 Habitat data

Water, meadow, and mudflat areas, as well as habitat heterogeneity, were selected as habitat factors. In this study, one image was selected for each water level phase to represent habitat changes. A total of nine remote sensing images were selected for interpretation and calculation of the areas of the three habitat types. Images were obtained from the official website of the European Space Agency (https://scihub.copernicus.eu/dhus/#/home), and the image class was Sentinel‐2 S2A. All acquired images had a cloud cover of less than 10%. Because Sentinel‐2 S2A images do not need to be preprocessed, ENVI 5.3 software was used to directly supervise their classification. The support vector machine method was used to classify habitats into water, meadows, and mudflats (Zhang, Zhang, et al., 2021; Zhang, Zhou, et al., 2021). The classification accuracy was tested based on reference field survey data, and the accuracy after testing reached over 90%. At the landscape scale, the Shannon's Diversity Index (SHDI) was selected to indicate habitat heterogeneity of Shengjin Lake at different water level stages (Kupfer, 2012) and calculated using Fragstats (Version 4.2).

#### 2.2.3 Waterbird data

Seven sampling sites were established in each sub‐lake for a total of 21 sampling sites. The waterbird surveys were conducted from October 2022 to March 2023. Three waterbird surveys were conducted during each water level period with an interval of 5 days between surveys. Waterbird surveys were conducted on days with clear weather; in the event of rain or fog, the surveys were postponed. Two teams worked simultaneously to avoid double counting, and each survey was completed within 1 day, from 07:00 to 17:00. Waterbird surveys were conducted using a telescope (ATS 80, 20–60×; Swarovski, Absam, Austria) within 1 km of the effective field of view. The survey time at each sampling point was 20 min (An et al., 2018). Direct counts were used for waterbird species with a small number of waterbirds, and group‐number counting methods were used for a large assemblage of waterbirds (Jia et al., 2018; Wang et al., 2020).

#### 2.2.4 Functional diversity

We selected 15 functional traits to calculate functional diversity. Because waterbirds play a significant role in ecosystems by accessing wetland resources, the traits selected are associated with food acquisition and foraging types. These traits included five morphological traits (beak, tarsus, wing, and tail lengths and hand‐wing index). Beak length and tarsus length represented the foraging ability of waterbirds, and wing length, tail length, and hand‐wing index represented the flight capability of waterbirds. We also included seven foraging traits and three foraging stratum traits (Table 1). Functional trait data were obtained from the Global Bird Trait Dataset (Tobias, 2022; Tobias et al., 2022). Gower's distance was used to calculate the functional distance between species based on trait values. This allowed the simultaneous use of categorical and numerical features to construct a distance matrix. The functional distance matrix was then subjected to principal coordinate analysis, in which the first two axes represented the new characteristic values. Functional diversity indices were calculated using the new characteristic values and species abundance data. Four functional diversity indices were selected that were independent and assessed different aspects of functional diversity: functional richness (FRic), functional evenness (FEve), functional divergence (FDiv), and functional dispersion (FDis). FRic measured the size of the functional space occupied by species within a community, reflecting the degree of utilization of ecological space. The larger the index, the higher the degree of ecological space utilization. FEve measured the evenness of the distribution of functional traits of species within the ecological space in a community. FDiv indicated the distinctiveness of species trait values within a community, reflecting the niche differentiation and resource competition among species, and the higher the index, the higher the degree of niche complementarity among species within the community (Villeger et al., 2008). FDis represented a measure of similarity between species within a community, with higher FDis reflecting high levels of niche differentiation (Laliberte & Legendre, 2010). Functional diversity indices were calculated using the “FD” package in R (Version 4.3.1).

**TABLE 1:** Functional characteristics used to calculate functional diversity in waterbird communities.

Functional type	Functional trait	Categories
Morphological traits	Beak length, Tarsus length, Wing length, Tail length, Hand‐Wing. Index	Continuous
Food types	Invertebrates, Mammals, Amphibians, Fish, Fruit, Seed, Plant	Binary
Pick‐up location	Below the water surface, Above the water surface, Ground	Binary

### 2.3 Statistical analysis

To compare the differences in richness, abundance, and functional diversity among different sub‐lakes, we first used the Shapiro–Wilk test to assess whether the data followed a normal distribution. Normally distributed data were analyzed using a one‐way ANOVA, whereas data that did not follow a normal distribution were analyzed using a Kruskal–Wallis test. These analyses were performed using the “vegan” package in R.

Multiple linear regression was used to fit the relationships between habitat and functional diversity. Before conducting model fitting, spatial autocorrelation analysis was performed using the Global Moran's I index. The Global Moran's I index showed *p* = .912, indicating the absence of spatial autocorrelation. Water, mudflat, meadow areas, and habitat heterogeneity index were selected as habitat factors. To avoid multicollinearity, we selected explanatory variables with a variance inflation factor (VIF) <5 for multiple regression modeling. First, all four habitat factors were included in the model for multicollinearity testing, and the habitat factor with the highest VIF value was gradually removed. Ultimately, the water area, mudflat area, and meadow area were selected as fixed variables for model fitting; the response variables were functional diversity indices. Models were screened according to the Akaike information criterion (AICc), and those with ΔAICc <2 were selected as candidate models (Grueber et al., 2011) and analyzed using the “MuMIn” package in R.

After understanding the relationship between the functional diversity of waterbird communities and habitat factors, we further investigated the specific relationships between functional traits and habitat factors by using a combined approach of the R‐mode linked to Q‐mode (RLQ) (Dolédec et al., 1996) and the trait–environment correlation matrix (fourth‐corner analyses) (Dray et al., 2014). RLQ corresponds to three different matrices, namely the environmental factor matrix (R), species abundance matrix (L), and species functional trait matrix (Q). RLQ aims to determine the relationship between environmental factors mediated by species abundance and traits, whereas fourth‐corner analysis was used to test the associations between specific species traits and environmental factors. First, each matrix was analyzed using different ordination methods. Correspondence analysis was used for the species abundance matrix. Principal component analysis was applied to the environmental factor and species functional traits matrices with numerical types as quantitative variables, as well as weighting according to species weight. Subsequently, RLQ analysis was performed, retaining two ordination axes in the results. Based on the RLQ ordination axes, fourth‐corner analysis was applied to test the relationship between environmental factors and specific functional traits. Given the involvement of 49,999 permutations, we used the Benjamini–Hochberg method to correct the *p* values and analyzed them using the “ade4” package in R (Dray & Dufour, 2007). All analyses were performed in R (4.3.1).

## 3 RESULTS

### 3.1 Wintering waterbird diversity and habitat changes during different water level phases

**FIGURE 3:**
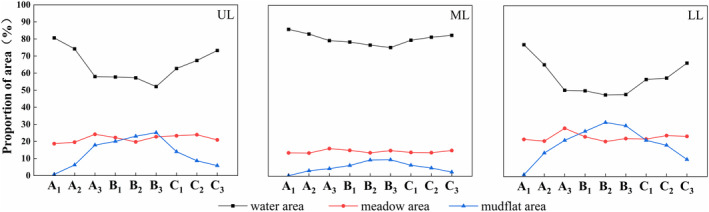
Habitat area changes in three sub‐lakes at different water level phases (A_1_: Early phase of decline, A_2_: Middle phase of decline, A_3_: Late phase of decline, B_1_: Early phase of stability, B_2_: Middle phase of stability, B_3_: Late phase of stability, C_1_: Early phase of rebound, C_2_: Middle phase of rebound, C_3_: Late phase of rebound). LL, lower lake; ML, middle lake; UL, upper lake.

During the declining water level phase, the proportion of water area in the UL and LL gradually decreased, whereas the proportion of mudflat area gradually increased. When the water level reached the A_3_ phase, the average water level was 10.74 m; the proportion of water area in the UL and LL decreased rapidly; and the proportion of mudflat area increased significantly, accompanied by the exposure of meadows. At this time, habitat differences among the three sub‐lakes were evident. During the water level stability phase, the average water level was 10.43 m, the lowest water level during the entire wintering period. The proportion of water area in the UL, LL, and ML was 55.68 ± 1.79%, 48.68 ± 0.76%, and 76.89 ± 0.92%, respectively. During the rebound water level phase, the proportion of water area in the UL and LL gradually increased, and the proportion of mudflat area gradually decreased. During the entire period of water level change, there were no significant changes in the proportion of meadows in the three sub‐lakes. The ML predominantly consisted of a water area, with a proportion of 80.47 ± 1.00%, whereas mudflat and meadow areas accounted for 4.82 ± 1.01% and 1.47 ± 0.37%, respectively (Figure 3).

The richness and abundance of waterbirds were higher in the UL and LL than in the ML throughout the period of water level change. As the water level declined, the richness and abundance of waterbirds in the UL, ML, and LL gradually increased. In the late phase of water level decline (A_3_), the average water level was 10.74 m, and the abundance of waterbirds in the three sub‐lakes increased rapidly. The lowest average water level recorded during the entire wintering period was 10.43 m. At this time, the abundance of waterbirds in the three sub‐lakes was the highest, with the UL and LL showing significant differences from the ML (UL vs. ML: *χ* ^*2*^ = 8.265, LL vs. ML: *χ* ^*2*^ = 6.861; all df = 1, *p* < .05). The average waterbird abundance in the UL, LL, and ML was 33,373.00 ± 899.40, 3107.67 ± 137.26, and 12,772.00 ± 1186.52, respectively. As the water level increased, the richness and abundance of waterbirds in the three sub‐lakes gradually decreased (Table 2).

**TABLE 2:** Wintering waterbird richness and abundance in three sub‐lakes of Shengjin Lake during different water level phases.

Index	Water level phase	Percentage of area of each habitat type (%)			Waterbirds richness(species)/abundance(individuals) in each sub‐lake		
		Water	Meadows	Mudflat	Upper Lake	Middle Lake	Lower Lake
Richness	A_1_	82.20	17.18	0.61	21.33 ± 3.40^a^	9.67 ± 0.94^a^	16.67 ± 1.89^a^
	A_2_	76.67	16.91	6.42	26.00 ± 2.16^a^	12.67 ± 2.05^a^	25.33 ± 4.11^a^
	A_3_	65.49	21.63	12.88	28.00 ± 2.94^a^	14.33 ± 1.70^b^	26.67 ± 2.62^a^
	B_1_	64.97	19.48	15.56	26.67 ± 1.25^a^	13.67 ± 0.94^b^	28.33 ± 0.47^a^
	B_2_	63.57	17.37	19.06	31.00 ± 1.41^a^	14.00 ± 0.00^b^	29.67 ± 1.70^a^
	B_3_	61.02	19.41	19.57	31.67 ± 1.25^a^	13.67 ± 0.47^b^	26.00 ± 0.82^a^
	C_1_	68.63	19.13	12.24	29.33 ± 0.47^a^	15.33 ± 0.47^b^	24.00 ± 0.82^a^
	C_2_	71.36	19.70	8.94	28.67 ± 0.47^a^	15.33 ± 0.47^a^	24.00 ± 0.82^a^
	C_3_	75.79	18.99	5.30	24.67 ± 0.94^a^	14.00 ± 0.82^a^	24.67 ± 1.25^a^
Abundance	A_1_	82.20	17.18	0.61	11488.33 ± 2526.60^a^	973.67 ± 135.55^a^	1688.00 ± 339.72^a^
	A_2_	76.67	16.91	6.42	17938.67 ± 3179.23^a^	1120.00 ± 179.29^a^	5551.33 ± 474.20^a^
	A_3_	65.49	21.63	12.88	28184.00 ± 1233.71^a^	1842.67 ± 277.81^b^	9876.00 ± 877.30^a^
	B_1_	64.97	19.48	15.56	30404.67 ± 1199.86^a^	2745.00 ± 133.64^b^	12524.67 ± 2269.68^a^
	B_2_	63.57	17.37	19.06	33373.00 ± 899.40^a^	3107.67 ± 137.26^b^	12772.00 ± 1186.52^a^
	B_3_	61.02	19.41	19.57	18134.33 ± 510.62^a^	3481.67 ± 98.06^b^	6974.33 ± 73.32^bc^
	C_1_	68.63	19.13	12.24	14244.33 ± 243.12^a^	2032.33 ± 75.46^b^	5845.33 ± 391.68^c^
	C_2_	71.36	19.70	8.94	8143.33 ± 435.76^a^	1769.33 ± 61.48^b^	5802.00 ± 81.03^a^
	C_3_	75.79	18.99	5.30	6061.00 ± 435.21^a^	1198.33 ± 99.39^a^	3512.00 ± 80.75^a^

^a–c^Different letters in the same row represent significant differences according to nonparametric analyses (*p* < .05).

### 3.2 Relationship between habitat factor and functional diversity under changing water level conditions

Multiple linear regression analysis indicated that the meadow area and mudflat area positively affected FRic, the meadow area positively affected FDis, whereas these variables were negatively affected by the water area. As the size of the water area increased, functional diversity decreased, and the functional characteristics of waterbirds tended to be concentrated (Table 3).

**TABLE 3:** Quantification of the relationship between habitat type and functional diversity via multiple linear regression.

Functional diversity	Models	X_1_	X_2_	X_3_	logLik	AICc	ΔAICc	Wi
FRic	1		0.341	−0.061	−8.731	31.2	0.00	0.684
	2	0.190	0.237	−0.086	−6.507	33.0	1.84	0.273
	3	0.442		−0.129	−11.631	37.0	5.80	0.038
	4		0.435		−16.171	41.3	10.17	0.004
	5	−0.183	0.500		−14.889	43.5	12.32	0.001
	6			−0.094	−19.203	47.4	16.23	0.000
FDis	1	0.051		−0.019	19.455	−25.2	0.00	0.820
	2	0.039	0.011	−0.017	21.039	−22.1	3.12	0.173
	3		0.033	−0.012	14.707	−15.7	9.50	0.007
	4			−0.015	7.774	−6.5	18.65	0.000

*Note*: The ΔAICc <2 is used as criteria for measuring the best model.

Abbreviations: FDis, functional dispersion; FRic, function richness; Wi, the model weights; X_1_, meadow area; X_2_, mudflat area; X_3_, water area.

**FIGURE 4:**
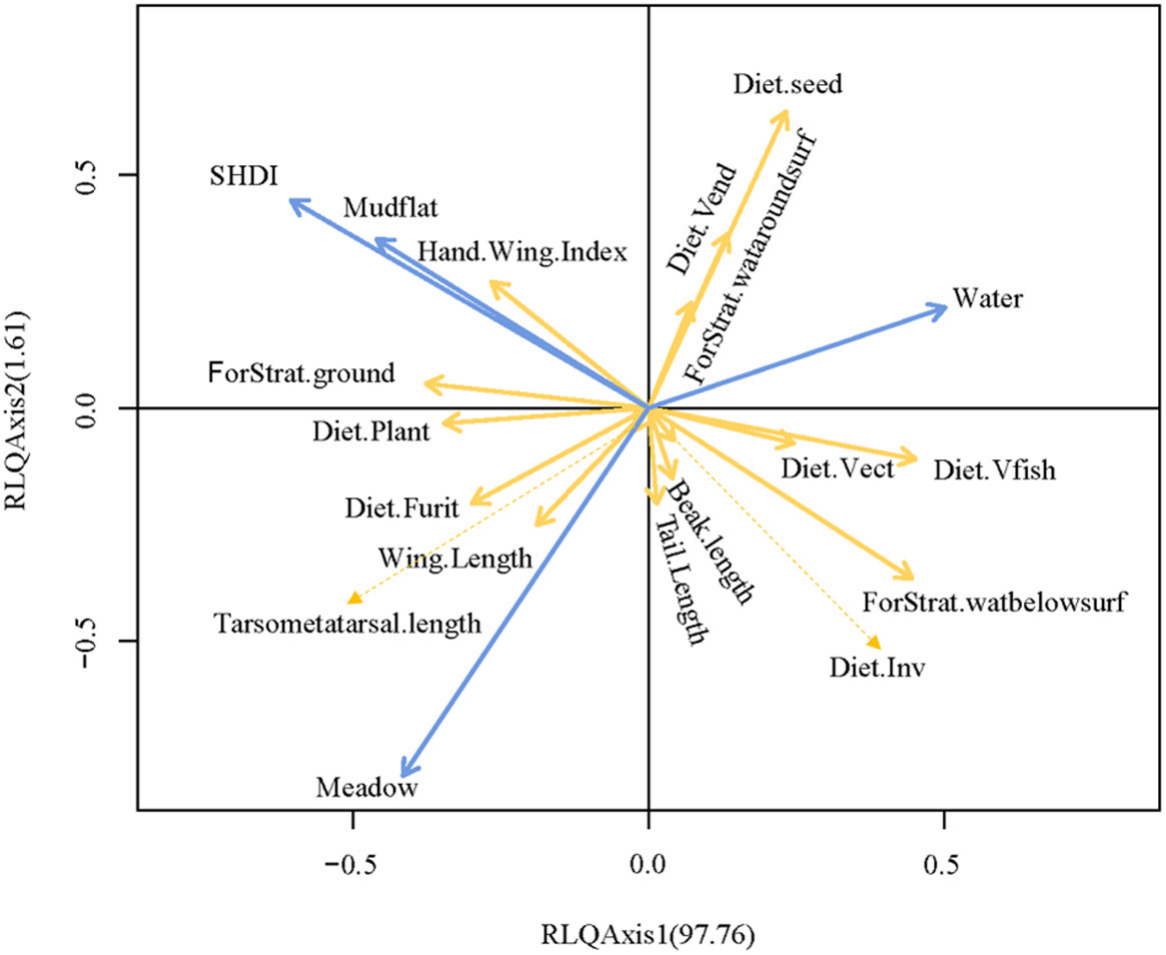
Results of RLQ analysis of habitat factors and functional traits of waterbirds. The figure indicates the correlation of habitat factors (blue arrows) and functional traits (yellow arrows) along the first two RLQ axes. The direction of the arrows represents the correlation between variables; arrows pointing in the same direction indicate a positive correlation, while arrows pointing in opposite directions indicate a negative correlation. Numbers in parentheses indicate the percentage of total co‐inertia accounted for by the first two RLQ axes. Correlations are mediated by waterbird abundance. SHDI, Shannon's Diversity Index. The full names of the other variables can be found in Appendix.

The RLQ analysis indicated that the first two axes explained 99.37% of the total covariance between habitat factors mediated by waterbird abundance and waterbird functional traits across the three datasets. There was a strong correlation between habitat factors and functional traits. The Hand Wing Index, ForStrat ground, DietPlant, Diet Fruit, Wing Length, Tarsometatarsal length, and SHDI, as well as Mudflat and Meadow, are all located on the left side of Axis 1, indicating a positive correlation between them. However, the remaining traits are situated on the right side of the axis, showing a positive correlation with water (Figure 4).

The integration of RLQ and the trait–environment correlation matrix (fourth‐corner analysis) revealed significant positive correlations between feeding on fish traits and water habitats and a significant negative correlation between mudflat habitats and the SHDI. The feeding on plant traits showed a significant positive correlation with the SHDI. The foraging layer below the water surface traits exhibited a significant positive correlation with water habitats but a significant negative correlation with mudflat and the SHDI. When the foraging layer was at the ground level, traits showed a significant positive correlation with mudflat habitats and the SHDI (Table 4).

**TABLE 4:** Fourth‐corner analysis of correlations between habitat factors and functional characteristics of waterbirds.

Test	Observed	*p* Value
Water/Diet.Vfish	0.34	.03
Mudflat/Diet.Vfish	−0.32	.04
SHDI/Diet.Vfish	−0.42	.01
SHDI/Dietplant	0.31	.04
Water/ForStrat.watbelowsurf	0.31	.01
Mudflat/ForStrat.watbelowsurf	−0.31	.02
SHDI/ForStrat.watbelowsurf	−0.46	.001
Mudflat/ForStratground	0.27	.03
SHDI/ForStratground	0.34	.02

*Note*: This table only lists variables that exhibit significant correlations (*p* < .05); correlations among other variables are presented in Table A1. Diet.Vfish indicates feeding on fish; Dietplant indicates feeding on plants; ForStrat.watbelowsurf indicates that the foraging layer is below the water surface; ForStratground indicates that the foraging layer is at ground level.

### 3.3 Differences in functional diversity of waterbirds in sub‐lakes at different water levels

**FIGURE 5:**
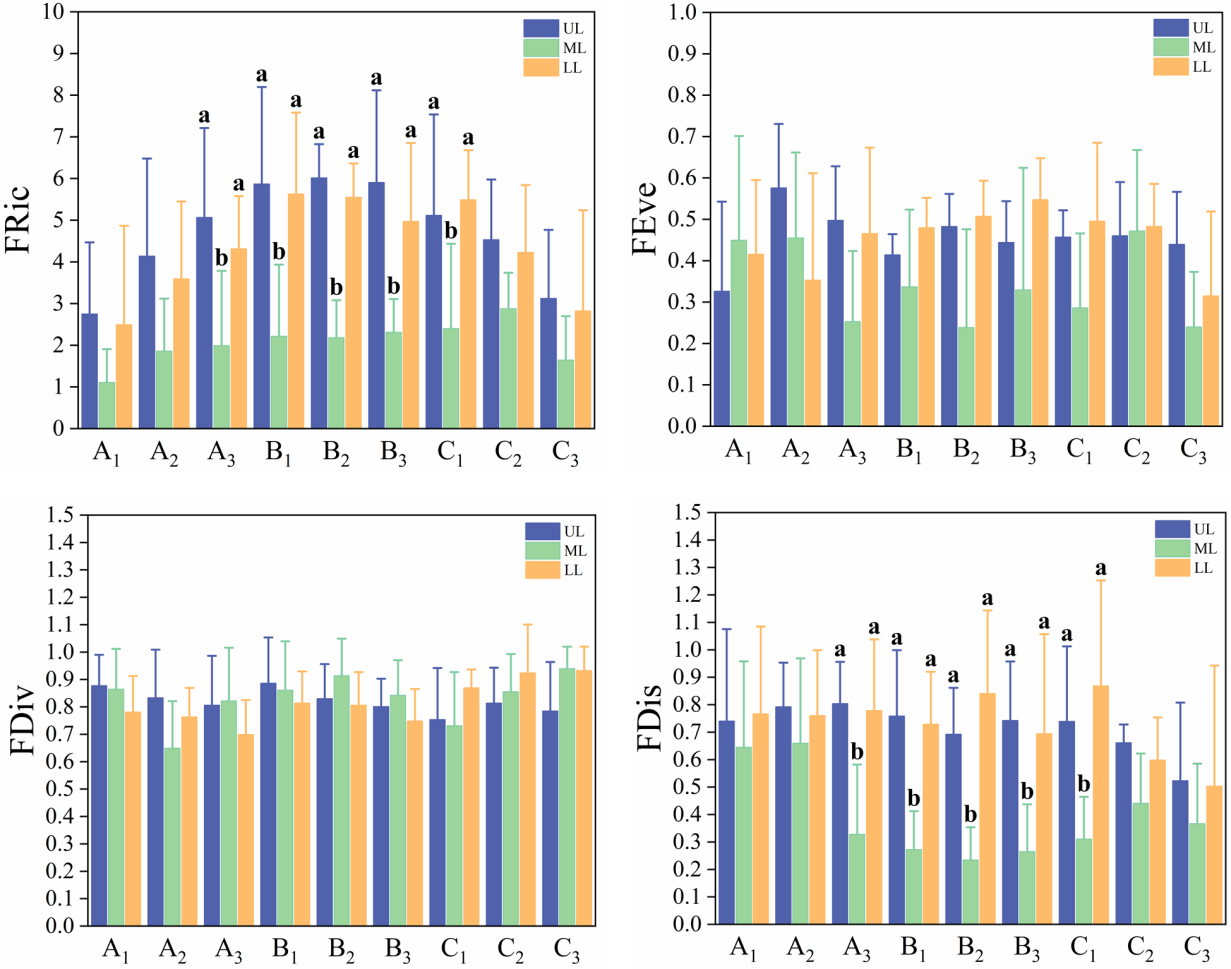
Comparison of functional diversity of three sub‐lakes at different water level phases (A_1_: Early phase of decline, A_2_: Middle phase of decline, A_3_: Late phase of decline, B_1_: Early phase of stability, B_2_: Middle phase of stability, B_3_: Late phase of stability, C_1_: Early phase of rebound, C_2_: Middle phase of rebound, C_3_: Late phase of rebound. FDiv, Functional divergence; FDis, Functional dispersion; FEve, Functional evenness; FRic, Functional richness; LL, lower lake; ML, middle lake; UL, upper lake). Different letters in the same water level phase indicate significant differences (*p* < .05). No letter in the same water level phase indicates no significant difference.

The functional diversity in the three sub‐lakes differed during the different water level phases. The FRic and FDis of the three sub‐lakes were not significantly different in the early and middle phases of water level decline. However, in the late phase of water level decline, the average water level was 10.74 m. The UL and LL showed significant differences in FRic and FDis compared with those of the ML (UL vs. ML: FRic: *χ* ^*2*^ = 5.011, FDis: *χ* ^*2*^ = 5.000; LL vs. ML: FRic: *χ* ^*2*^ = 5.600, FDis: *χ* ^*2*^ = 6.209; all df = 1, *p* < .05). At this water level phase, the FRic of the UL and LL increased rapidly. During the stable water level phase, the average water level was as low as 10.43 m, which was the lowest throughout the wintering period. At this time, the FRic and FDis of the three sub‐lakes reached their maximum values. The FRic and FDis of the UL and LL significantly differed compared with those of the ML (UL vs. ML: FRic: *χ* ^*2*^ = 9.822, FDis: *χ* ^*2*^ = 9.016; LL vs. ML: FRic: *χ* ^*2*^ = 9.822, FDis: *χ* ^*2*^ = 7.547; all df = 1, *p* < .05). During the early phase of water level rebound, the water level was 10.53 m, and FRic and FDis of the UL and LL significantly differed from those of the ML (UL vs. ML: FRic: *χ* ^*2*^ = 6.861, FDis: *χ* ^*2*^ = 8.265; LL vs. ML: FRic: *χ* ^*2*^ = 5.588, FDis: *χ* ^*2*^ = 5.588; all df = 1, *p* < .05). However, during the middle and late phases of water level rebound, there were no significant differences in functional diversity among the three sub‐lakes. Throughout the water level change period, there were no significant differences in FDiv and FEve among the three sub‐lakes (Figure 5).

## 4 DISCUSSION

### 4.1 Habitat differences in sub‐lakes at different water level phases

Differences in lake terrain and topography can affect habitat distribution patterns. Generally, areas with low lake beds are deep‐water habitats where environmental filtering selects for overlapping or similar functional features, leading to the formation of simpler waterbird communities (Almeida et al., 2018). By contrast, areas with high lake beds have more diverse habitats, including mudflats, meadows, shallow water, and deep‐water habitats. Mudflats are easily exposed in areas with high lake beds, providing suitable habitats for benthic and aquatic vegetation and leading to a high abundance and availability of food resources. Consequently, the waterbird species found in such areas are more diverse and exhibit higher functional diversity (Frota et al., 2022). Therefore, different regions of the lakes had different waterbird community structures. Consistent with our first hypothesis, there were significant differences in the functional diversity of waterbird communities among the areas of Shengjin Lake, with the UL and LL having higher functional diversity than the ML.

During phases of high water levels, all parts of the lake had similar habitat types dominated by water habitats. In such habitats, waterbirds that feed in the benthic zone and via carex‐feeding cannot obtain sufficient food resources (Li et al., 2020), which has a particularly adverse effect on waterbirds that migrate to wintering grounds (Yu et al., 2017). Mudflats are the optimal habitat for waterbirds, such as shorebirds, to forage (Aarif et al., 2020). Therefore, lower water levels are better for waterbird foraging because, as the water level decreases, the mudflat area gradually increases. During our survey, we observed that shorebirds responded positively to mudflat exposure, as the best time to acquire food was when the mudflats were not compacted (Yu et al., 2023). During the stable water level phase, the water level decreased to its lowest level during the entire wintering period. In addition to the gate‐controlled effects, strong evaporation and reduced rainfall led to a decrease in the water surface area and an increase in mudflats and meadows. At this time, all parts of the lake contained various habitat types, and the heterogeneity of habitats was the highest, which fulfilled the habitat requirements for different species of waterbirds (Kushlan, 2007). During the water level rebound phase, the water area increased, but the mudflat and meadow area decreased, reducing the abundance and availability of food resources and leading to a decrease in the quality of waterbird habitats. This can result in waterbirds not having sufficient energy reserves for migration (Aharon‐Rotman et al., 2017).

### 4.2 Effect of habitat type on functional diversity

Habitat types dominated by water habitats tend to be homogenous and have a low availability of food resources, negatively affecting the foraging of waterbirds (Li et al., 2020). Habitat filtering effects are evident, leading to the continuous presence and aggregation of species with specific functional traits in a small space, decreasing functional diversity (Li, Zhang, et al., 2019). The dominant species consist of waterbirds that feed on fish and forage below the water surface (Almeida et al., 2017). A decline in the water level can lead to habitat diversification and increased habitat heterogeneity, resulting in increased waterbird richness and abundance. However, species richness is positively related to FRic (Ding et al., 2013). Therefore, increased waterbird richness leads to increased FRic, suggesting that higher habitat heterogeneity not only accommodates more species but also encompasses higher functional diversity (Robichaud & Rooney, 2017). Additionally, different waterbirds use different foraging methods and select suitable habitats according to their physical characteristics. Therefore, diverse habitats can fulfill the requirements of different species of waterbirds and have a significantly positive effect on functional diversity (Almeida et al., 2017). Shorebirds that forage on the ground prefer mudflat habitats and exhibit different bill morphology, tarsal lengths, and feeding models (Zhang, Zhang, et al., 2021; Zhang, Zhou, et al., 2021). As mudflat areas increase, more food resources are available, attracting different species of waterbirds to forage. Therefore, mudflat habitats have a positive effect on functional diversity. Meadows provide the main food resources for waterbirds, such as the Bean Goose (*Anser fabalis*).

### 4.3 Functional diversity of sub‐lakes at different water level phases

Consistent with our third hypothesis, the functional diversity of the waterbird communities in the three sub‐lakes was the highest, and the differences were significant when the water level was at its lowest. The method based on measuring functional traits can effectively enhance the understanding of ecosystem functions. The different functional groups exhibited unique foraging strategies and morphological characteristics. Water level changes directly affect the abundance and availability of food resources, affecting the composition of functional groups within waterbird communities (Bolduc & Afton, 2008). Under the influence of natural hydrological rhythms, lake landscapes exhibit a high degree of spatiotemporal heterogeneity and diverse habitat types. Mudflat and meadow habitats are exposed to varying degrees during different wintering phases, providing a continuous food resource for different functional groups of waterbirds (Kingsford et al., 2004). Swimmers and waders are the main groups of wintering waterbirds. As the water level naturally falls, fish and other food resources can be concentrated in limited low‐lying areas, thereby increasing the availability of food resources (Bancroft et al., 2002). During the water level decline and rebound phases, all parts of the lake were dominated by water habitats, decreasing FDis. The functional traits of the waterbird community are similar, and competition increases, indicating a reduction in the abundance of waterbirds with extreme trait values. Under such habitat conditions, a strong habitat filtering effect is produced. Only species with specific functional traits can inhabit these areas, and they are unable to effectively occupy functional spaces, leading to functional redundancy and convergent functional traits in the waterbird community (Henry & Cumming, 2017). For example, Little Grebes (*Tachybaptus ruficollis*) mainly inhabit such habitats, and their food resource utilization and morphological characteristics are similar, resulting in insignificant differences in functional diversity among waterbird communities in water habitats. FRic represents the extent to which species occupy functional spaces within a community, and higher FRic indicates that waterbirds can effectively occupy functional spaces (Almeida et al., 2020). During the stable water level phase, the water level was the lowest; this phenomenon exposed many mudflats and meadows, attracting different species of waterbirds to forage, increasing the functional characteristic values of waterbird species, and expanding the spatial range of waterbird communities (Almeida et al., 2019), increasing FRic. The high degree of FDis reflects a high level of niche differentiation, enhancing complementarity in niches and allowing for more thorough resource usage within the community (Prada‐Salcedo et al., 2021). Compared with that when the water level was declining, the FDis of waterbirds during the stable water level phase was higher, and the difference was significant.

## 5 CONCLUSIONS

This study explored the process of waterbird community assembly based on functional diversity, provided an in‐depth analysis of the impact that gate‐controlled activities have on waterbird communities in different areas, and formulated a reasonable water level regulation scheme for Shengjin Lake. Our results highlighted the dominant role of habitat filtering of particular traits in waterbird community assembly. When the water level of Shengjin Lake fluctuated between 10.43 and 10.74 m, exposing mudflats and meadows and increasing the heterogeneity of habitats, the needs of waterbirds with different functional traits were satisfied. This can act as compensation during the peak of waterbird migration under high‐intensity competition and maintain the high functional diversity of the waterbird community, promoting the differentiation of waterbird functional traits. The ML predominantly comprised water habitats with evident habitat filtering, where only piscivorous waterbirds and waterbirds that forage below the water surface were observed. This led to a lack of functional traits, resulting in significantly lower functional diversity in the ML than in the UL and LL.

In summary, we suggest that the water level of Shengjin Lake should decline sequentially throughout the wintering period to allow for increased abundance and availability of food resources. The minimum water level regulation range for the entire wintering period should be between 10.43 and 10.74 m. Under such conditions, the habitat types would be more diverse and be able to sustain a higher functional diversity of waterbirds, maximizing the functioning of the ecosystem. This study has scientific implications for gate‐controlled activities at Shengjin Lake based on waterbird conservation. However, for lakes with less abundant food resources, maintaining a hydrological status for a long period of time may not be conducive to the maintenance of waterbird diversity, and this should be given attention in future research.

## AUTHOR CONTRIBUTIONS

**Yongzhi Wang:** Data curation (equal); formal analysis (equal); investigation (equal); methodology (equal); software (equal); validation (equal); visualization (equal); writing – original draft (equal); writing‐ review and editing (equal). **Xianglin Ji:** Conceptualization (equal); formal analysis (equal); methodology (equal); software (equal); writing – original draft (equal); writing – review and editing (equal). **Lizhi Zhou:** Conceptualization (equal); funding acquisition (equal); resources (equal); supervision (equal); writing – review and editing (equal).

## FUNDING INFORMATION

This research was funded by the National Natural Science Foundation of China (No. 32171530).

## CONFLICT OF INTEREST STATEMENT

The authors declare no conflict of interest.

## Supporting information


Data S1.

## Data Availability

Data are provided as Data S1.

## Appendix

**TABLE A1:** Fourth‐corner analysis of correlations between habitat factors and functional characteristics of waterbirds.

Test	Observed	*p* Value
water/Mouth.peak	0.041	.865
mud/Mouth.peak	−0.074	.662
grass/Mouth.peak	0.005	.999
SHDI/Mouth.peak	−0.01	.999
water/Tarsometatarsal.length	−0.002	.956
mud/Tarsometatarsal.length	−0.023	.933
grass/Tarsometatarsal.length	0.031	.815
SHDI/arsometatarsal.length	0.053	.670
water/Wing.Length	−0.139	.434
mud/Wing.Length	0.085	.588
grass/Wing.Length	0.163	.329
SHDI/Wing.Length	0.179	.347
water/Tail.Length	0.016	.926
mud/Tail.Length	−0.051	.736
grass/Tail.Length	0.029	.883
SHDI/Tail.Length	−0.002	.981
water/Hand.Wing.Index	−0.193	.266
mud/Hand.Wing.Index	0.213	.110
grass/Hand.Wing.Index	0.123	.500
SHDI/Hand.Wing.Index	0.255	.165
water/DietInv	0.026	.905
mud/DietInv	−0.030	.860
grass/DietInv	−0.018	.939
SHDI/DietInv	−0.050	.826
water/Diet.Vend	0.126	.502
mud/Diet.Vend	−0.085	.602
grass/Diet.Vend	−0.136	.441
SHDI/Diet. Vend	−0.071	.751
water/Diet.Vect	0.189	.280
mud/Diet.Vect	−0.188	.183
grass/Diet.Vect	−0.140	.400
SHDI/Diet.Vect	−0.218	.250
water/DietVfish	0.337	.025
mud/DietVfish	−0.319	.039
grass/DietVfish	−0.265	.095
SHDI/DietVfish	−0.421	.010
water/Diet.Furit	−0.235	.058
mud/Diet.Furit	0.197	.114
grass/Diet.Furit	0.218	.081
SHDI/Diet.Furit	0.251	.093
water/Diet.seed	0.203	.248
mud/Diet.seed	−0.117	.456
grass/Diet.seed	−0.242	.102
SHDI/Diet.seed	−0.156	.451
water/DietPlant	−0.262	.084
mud/DietPlant	0.235	.115
grass/DietPlant	0.221	.106
SHDI/DietPlant	0.314	.044
water/ForStrat.watbelowsurf	0.311	.017
mud/ForStrat.watbelowsurf	−0.310	.020
grass/ForStrat.watbelowsurf	−0.228	.126
SHDI/ForStrat.watbelowsurf	−0.460	.001
water/ForStratwataroundsurf	0.077	.684
mud/ForStratwataroundsurf	−0.066	.675
grass/ForStratwataroundsurf	−0.072	.695
SHDI/ForStratwataroundsurf	−0.015	.978
water/ForStratground	−0.286	.058
mud/ForStratground	0.276	.034
grass/ForStratground	0.224	.143
SHDI/ForStratground	0.335	.016

*Note*: SHDI indicates Shannon's Diversity Index; water indicates water habitat; mud indicates mudflat habitat; grass indicates meadow habitat; Diet.Vfish indicates feeding on fish; Dietplant indicates feeding on plants; Diet.Furit indicates feeding on furit; Diet. Vend indicates feeding on mammals; Diet.Vect indicates feeding on amphibians; Diet.seed indicates feeding on seed; ForStrat.watbelowsurf indicates that the foraging layer is below the water surface; ForStratground indicates that the foraging layer is at ground level; ForStratwataroundsurf indicates that the foraging layer is the water surface.
